# A Phase I Study of the Combination of Temsirolimus with Irinotecan for Metastatic Sarcoma

**DOI:** 10.3390/cancers5020418

**Published:** 2013-04-11

**Authors:** Claire F. Verschraegen, Sujana Movva, Yongli Ji, Berndt Schmit, Robert H. Quinn, Ben Liem, Therese Bocklage, Monte Shaheen

**Affiliations:** 1 Department of Hematology/Oncology, The University of Vermont Cancer Center, 89 Beaumont Ave., Burlington, VT 05405, USA; 2 Department of Medical Oncology, Fox Chase Cancer Center, Pennsylvania, PA 19111, USA; 3 Department of Radiology, University of Pittsburgh Medical Center East, Pittsburgh, PA 15146, USA; 4 Department of Orthopaedics, University of Texas Health Science Center San Antonio, San Antonio, TX 78229, USA; 5 Department of Radiation Oncology, University of New Mexico Cancer Center, Albuquerque, NM 87131, USA; 6 Department of Pathology, University of New Mexico Cancer Center, Albuquerque, NM 87131, USA; 7 Division of Hematology/Oncology, University of New Mexico Cancer Center, Albuquerque, NM 87131, USA

**Keywords:** sarcoma, mTOR inhibitors, irinotecan, temsirolimus, phase I

## Abstract

mTOR inhibitors are emerging as important anti-neoplastic agents with a wide range of clinical applications. The topoisomerase I inhibitor irinotecan is a potent DNA damaging drug, with a broad spectrum of anticancer activities. mTOR appears to enhance cancer cell survival following DNA damage, thus the inhibition of mTOR after irinotecan could theoretically show synergistic activities in patients. Both mTOR inhibitors and irinotecan have been used as single agents in soft tissue sarcomas with limited efficacy. We completed a phase I trial of the combination of the mTOR inhibitor, temsirolimus, and irinotecan in patients with advanced soft tissue sarcoma. Seventeen patients were recruited. The Phase II recommended dose is 20 mg of temsirolimus and 80 mg/m^2^ of irinotecan administered on weekly basis for three out of four weeks. Most frequently encountered toxicities include cytopenias, fatigue, and gastrointestinal toxicities. Two patients (one with leiomyosarcoma and one with high grade undifferentiated sarcoma) had stable disease for more than 12 months.

## 1. Introduction

**Figure 1:**
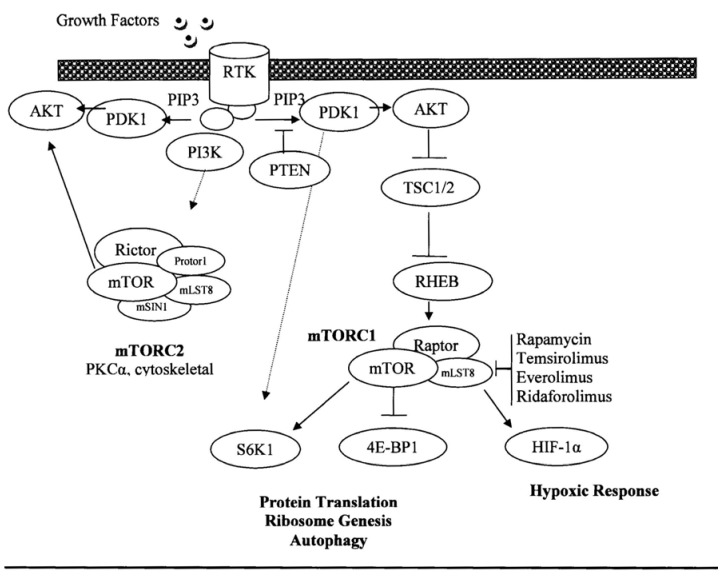
mTOR pathway.

The mammalian target of rapamycin (mTOR) is an essential signaling protein that has an important role in various cellular processes, particularly in nutrient sensing, cell mass regulation and proliferation [1]. mTOR operates downstream from the PI3K signaling pathway, which is frequently up-regulated in cancer [2]. mTOR is an atypical serine/threonine protein kinase of the phosphatidylinositol 3-kinase (PI3K)-related kinase family, which functions by complexing with other proteins (mTOR complex 1 and 2) to promote protein translation through phosphorylation of the translational regulators, eukaryotic translation initiation factor 4E (eIF4E)-binding protein 1 (4E-BP1) and S6 kinase 1 (S6K1) (Figure 1) [1,3]. mTOR also up-regulates the expression of hypoxia inducible factor (HIF)-1α, which mediates the expression of several key angiogenic factors including the vascular endothelial growth factor. Thus, inhibition of mTOR also results in an antiangiogenic effect in sarcomas [4,5]. Additionally, the loss of p53, a common event in cancer, promotes mTOR activation [6]. Multiple familial cancer syndromes occur due to mutations in genes encoding proteins that signal upstream of the mTOR complexes, (including tuberous sclerosis proteins 1 and 2 (TSC1/2*)*, serine threonine kinase 11 (STK11 or Lkb1), phosphatase and tensin homolog (PTEN), and neurofibromatosis type 1 (NF1). Oncogenic activation of mTOR induces several processes required for cancer cell growth, survival, and proliferation [1]. Thus, inhibition of mTOR kinase activity impacts multiple pathways that are important for tumor maintenance such as the protein translation of several oncogenes [3]. 

Two mTOR inhibitors, analogs of rapamycin, temsirolimus (Torisel^®^) and everolimus (RAD-001, Afinitor^®^) are clinically available for renal cell cancer [7,8,9], well differentiated neuroendocrine tumors [10], and in combination with hormonal therapy for metastatic breast cancer [11]. mTOR inhibitors have modest inhibitory activity against soft tissue sarcoma except in perivascular epithelioid cell tumors [12] and angiomyolipomas [13]. In a phase II study of temsirolimus in patients with untreated advanced soft tissue sarcoma, two patients (with undifferentiated fibrosarcoma and uterine leiomyosarcoma) achieved a confirmed partial response lasting 3 and 17 months, respectively [14]. A phase II study of ridaforolimus in patients with refractory sarcoma also showed a potential clinical benefit [15]. Ridaforolimus, as maintenance therapy after chemotherapy, was tested in a phase III trial in patients with soft tissue sarcoma. A clinical benefit rate of 29% was observed in the 212 evaluable patients with a minor survival advantage [16]. Most common adverse events are anemia, hyperglycemia, dyspnea, nausea and vomiting, neutropenia, hypokalemia, fatigue and stomatitis [17]. 

Irinotecan is a topoisomerase I inhibitor that generates DNA damage in replicating cells [18], and triggers cell cycle arrest, apoptosis, and senescence [19]. Single agent and various combinations of irinotecan have been used as salvage therapies for a variety of pediatric soft tissue sarcomas with responses varying from 15 to 50% [20,21,22,23]. The pharmacokinetics of topoisomerase inhibitors are highly variable, thus not reliable and not helpful for defining the optimal schedule. Animal experimentation shows that prolonged exposure is better than high doses intermittently, thus the choice of a weekly dosing for this study [24]. 

Cancer cell survival following irinotecan-induced DNA damage is regulated by mTOR. A synergy was found between irinotecan and rapamycin in human xenografts colon cancers. mTOR interferes with hypoxia-inducible factor-1α (HIF-1α), a key transcription factor with a pivotal role in tumor cell metabolism (Figure 1). Topoisomerase I inhibitors, such as irinotecan, also inhibits the accumulation of HIF-1α. Human colon cancers xenografted in nude mice treated with the combination of low doses irinotecan and rapamycin showed a potent inhibition of the mTOR/HIF-1α axis, which was accompanied by a dramatic reduction in tumor volume, compared to single agent. *In vitro* experiments further confirmed this synergy [25]. These results identify HIF-1α as the potential target and provide a rationale for this study testing the combination of irinotecan and temsirolimus in soft tissue sarcomas. We hypothesized that mTOR inhibition could potentiate the clinical activity of irinotecan by preventing the regulation of cancer cell survival. The primary objective of this study was to determine the maximum tolerated dose (MTD) and the toxicity profile of the combination. A secondary objective was to evaluate the clinical activity of this regimen. 

## 2. Experimental Section

The study (NCT00996346) was approved by the Institutional Review Board and all patients signed an informed consent before enrollment. This trial was conducted according to Good Clinical Practice guidelines. Eligibility criteria included patients older than 17 years, with a histologically proven advanced soft tissue sarcoma, and who had failed at least one prior treatment for metastatic disease. Recurrence had to be assessable by RECIST criteria version 1. Patients had to have a performance status of 0–2 and normal organ function (peripheral granulocyte count of equal or greater than 1,500 cells/mm^3^, hemoglobin equal or greater than 8 g/dL, platelet count equal or greater than 100,000/mm^3^ and absence of a regular red blood cell transfusion requirement; normal hepatic function with a total bilirubin equal or lower than the upper limit of normal and SGOT or SGPT equal or lower than two times the upper limit of normal, adequate renal function defined by a serum creatinine equal or lower than 1.5 times the upper limit of normal, and a clinically normal cardiac function) with a fasting total cholesterol and triglyceride levels below 350 mg/dL and 400 mg/dL, respectively. Pregnant women or nursing mothers were not eligible. 

The study was planned to be conducted in two parts. A 3+3 phase I two-arm crossing design was utilized in Part 1 to define the MTD [26]. A part 2 of the study (Expansion phase) was planned if two or more objective responses were observed during the part 1 after recruiting 17 patients, but was never implemented. Part 1 was done in two arms using a fixed dose of one drug in each arm [26]. Both drugs were administered on a weekly basis for three consecutive doses, followed by one week of rest. Irinotecan was given first over 60 min, followed by temsirolimus over 30 min. Each cycle lasted four weeks. No intra-patient dose escalations were allowed. Each patient was treated until disease progression or intolerable side effects developed. Arm A consisted of a fixed dose of irinotecan at 80 mg/m^2^ and a starting dose of temsirolimus of 15 mg with increments of 5 mg for subsequent cohorts. Arm B consisted of a fixed dose of temsirolimus 25 mg and a starting dose of irinotecan of 50 mg/m^2^ with increments of 15 mg/m^2^ for subsequent cohorts.

DLTs were defined as grade 3 neutropenia on retreatment day, a grade 4 febrile neutropenia, a drug-related grade 3 or 4 non-hematologic toxicity (except fatigue, nausea, vomiting or grade 3 hypersensitivity reaction), a grade 2 or greater motor or sensory neuropathy, or inability to receive consecutive doses of treatment during the first four weeks of treatment. Adverse events were assessed with the NCI Common Terminology Criteria for Adverse Events, Version 3. The MTD was defined as the dose preceding that at which at least two out of six patients experience a DLT. For patients’ safety, doses of the fixed drug could be reduced by one tier in subsequent cycles, after the first cycle was appropriately completed. Statistics were descriptive for part 1. Repeated cycles at the same dose level were given to patients who benefited from treatment (complete or partial remission, or stabilization of disease) after resolution of non-hematologic toxicity to a grade 0 or 1, and return of absolute neutrophil count to ≥1,500 cells/mm^3^ and platelet count to ≥100,000/mm^3^. If toxic effects precluding therapy persisted for three weeks or more, patients were removed from the study. Patients were examined every four weeks and blood parameters were verified weekly. Imaging studies were repeated every eight weeks and tumors evaluated by the RECIST criteria version 1. The clinical benefit rate was calculated as the sum of complete and partial responses plus stable disease.

## 3. Results and Discussion

Thirty-five patients were screened and 17 patients were enrolled between October 2009 and May 2011. Reasons for enrollment failure were ineligibility (16 patients) and refusal to participate (two patients). Patient characteristics are described in Table 1. 

**Table 1:** Patient characteristics (N = 17).

**Median Age**		57 years (range, 26–72)
**Performance Status**		0 (0–2)
**Races/Ethnicities**	Non-Hispanic White	8
	Hispanic White	7
	Black	2
		Male/Female
**Histology**	Undifferentiated sarcoma	4/1
	Leiomyosarcoma	0/4
	Liposarcoma	1/2
	Myxofibrosarcoma	3/0
	Peripheral Nerve Sheath Tumor	1/0
	Extraosseous osteosarcoma	1/0
**`Prior Therapies**	Surgery	14
	Radiotherapy	8
	One prior chemotherapy	13
	More than one chemotherapy	4

Three patients were enrolled on Arm B (fixed dose of temsirolimus 25 mg and a starting dose of irinotecan of 50 mg/m^2^). All three patients had a dose limiting toxicity (DLT); grade 3 neutropenia on retreatment day 8 in one patient, and inability to receive three doses of weekly treatment in two patients who described gastrointestinal symptoms of nausea, emesis, and diarrhea. One of these two patients received the first dose and refused further administrations. He also had a DLT of grade 3 muscle weakness, related to electrolyte imbalance from the gastrointestinal symptoms. The other patient had a second administration at a reduced dose of temsirolimus from 25 mg to 20 mg, and then refused the third dose. Thus this arm was closed. Arm A (fixed dose of irinotecan at 80 mg/m^2^ and a starting dose of temsirolimus of 15 mg) was completed after fourteen patients were accumulated, six at the starting level and eight at the next dose level (fixed dose of irinotecan at 80 mg/m^2^ and temsirolimus at 20 mg). There was one patient with a DLT at the starting dose who did not recover the platelet count to 100,000/mm^3^ at the end of the 4th week. Thus, three additional patients were treated at that level and no further DLT were observed. The second cohort was the last cohort since 25 mg per week of temsirolimus could not be combined with a lower dose of irinotecan in Arm B. A total of eight patients were enrolled at that level. No DLTs were observed. Because no objective responses were seen in these patients, this regimen was not sufficiently active in refractory soft tissue sarcoma and part 2 of the study was never initiated. Adverse events are described in Table 2. The hematological side effects were manageable, usually a grade 1 or 2 in a third of the patients, but timely recovery of blood counts did not always happen with additional cycles of therapy, indicating a possible cumulative toxicity. Other side effects included gastrointestinal intolerance with nausea, diarrhea and vomiting, again in a third of the patients, as well as fatigue and skin rash. 

**Table 2:** Adverse events per cohort for all cycles of treatment.

Adverse Event	Cohort	Grade 1	Grade 2	Grade 3	Grade 4	Total/cohort	Percentage for all patients
Anemia	Arm B level 1 (N = 3)						24%
	Arm A level 1 (N = 6)	1				1	
	Arm A level 2 (N = 8)		3			3	
Neutropenia	Arm B level 1 (N = 3)		1	*1*		2	35%
	Arm A level 1 (N = 6)	1	1			2	
	Arm A level 2 (N = 8)		1	1		2	
Thrombocytopenia	Arm B level 1 (N = 3)	1				1	29%
	Arm A level 1 (N = 6)	1	1			2	
	Arm A level 2 (N = 8)	2				2	
Fever	Arm B level 1 (N = 3)					1	12%
	Arm A level 1 (N = 6)	1		1 ^§^		1	
	Arm A level 2 (N = 8)						
Abdominal pain	Arm B level 1 (N = 3)		1			1	12%
	Arm A level 1 (N = 6)	1				1	
	Arm A level 2 (N = 8)						
Anorexia	Arm B level 1 (N = 3)		2			2	12%
	Arm A level 1 (N = 6)						
	Arm A level 2 (N = 8)						
Diarrhea	Arm B level 1 (N = 3)	1	1			2	35%
	Arm A level 1 (N = 6)		1			1	
	Arm A level 2 (N = 8)	2	1			3	
Nausea	Arm B level 1 (N = 3)		2			2	35%
	Arm A level 1 (N = 6)	2				2	
	Arm A level 2 (N = 8)	1	1			2	
Vomiting	Arm B level 1 (N = 3)	1	1	1		3	24%
	Arm A level 1 (N = 6)						
	Arm A level 2 (N = 8)	1				1	
Increased transaminases	Arm B level 1 (N = 3)						6%
	Arm A level 1 (N = 6)						
	Arm A level 2 (N = 8)	1				1	
Mucositis	Arm B level 1 (N = 3)						12%
	Arm A level 1 (N = 6)	1	1			2	
	Arm A level 2 (N = 8)						
Fatigue	Arm B level 1 (N = 3)		1	1		2	35%
	Arm A level 1 (N = 6)	2			1	3	
	Arm A level 2 (N = 8)		1			1	
Headache	Arm B level 1 (N = 3)		1			1	18%
	Arm A level 1 (N = 6)		1			1	
	Arm A level 2 (N = 8)	1				1	
Rash	Arm B level 1 (N = 3)	2				2	29%
	Arm A level 1 (N = 6)						
	Arm A level 2 (N = 8)	2	1			3	
Hyperglycemia	Arm B level 1 (N = 3)	1				1	12%
	Arm A level 1 (N = 6)		1			1	
	Arm A level 2 (N = 8)						
Muscle weakness	Arm B level 1 (N = 3)			*1*		1	6%
	Arm A level 1 (N = 6)						
	Arm A level 2 (N = 8)						
Sensory neuropathy	Arm B level 1 (N = 3)						6%
	Arm A level 1 (N = 6)						
	Arm A level 2 (N = 8)	1				1	
Thrombosis	Arm B level 1 (N = 3)						6%
	Arm A level 1 (N = 6)			1 ↑		1	
	Arm A level 2 (N = 8)						
Not recovering counts on re-treatment day or inability to receive a full cycle of treatment on time	Arm B level 1 (N = 3)					*3*	100%
	Arm A level 1 (N = 6)					*1*	18%
	Arm A level 2 (N = 8)						

Dose limiting toxicities occurring during the first cycle only are noted in *italics*; ^§^, neutropenic fever at third cycle; ↑, at 5th cycle.

There were no objective responses. However, two patients who had progressive disease prior to entry into the study stabilized their disease for 12 cycles each. One had a leiomyosarcoma, and the other was diagnosed with an undifferentiated grade 3 sarcoma and is the only patient who remains alive two years later. Two more patients, one with a liposarcoma and one with an undifferentiated sarcoma, received four cycles each. All other patients progressed after two cycles. 

## 4. Conclusions

Disruption of PI3K-AKT-mTOR pathways has been associated with different sarcoma subtypes. Rapamycin and its analogs, which bind to the intracellular 12-kDa FK506-binding protein (FKBP-12) to form a protein-drug complex, inhibit the activity of mTOR [27,28,29,30]. The growth of multiple sarcoma cell lines, including rhabdomyosarcoma [31], osteosarcoma cell lines [32], and Ewing sarcoma [33], is inhibited by the rapamycin analogs. A multicenter phase II study of rapamycin in patients with soft tissue sarcoma did not appear to be effective when administrated as a monotherapy, although one patient with fibrosarcoma achieved a partial response after two cycles lasting at least 36 weeks [34]. Subsequently, ridaforolimus showed promising efficacy in various trials enrolling patients with advanced sarcoma [15,35], but as an indication for the maintenance of remission in patients treated for metastatic sarcoma it was not approved because of the failure of the drug to exhibit a meaningful clinical activity [36]. In combination, the mTOR inhibitor, sirolimus, with cyclophosphamide is tolerated by the majority of sarcoma patients with a clinical benefit rate of about 20% [37].

In this study, we attempted to capitalize on *in vitro* data suggesting that mTOR inhibition could enhance tumor cell killing by DNA damaging agents such as irinotecan through the HIF-1α pathway. The aim of this study was to identify the MTD of the combination of irinotecan and temsirolimus in patients with refractory sarcomas, followed by expansion of this patients’ cohort to probe clinical activity. We did identify a dose of irinotecan of 80 mg/m^2^ and temsirolimus of 20 mg weekly for three consecutive weeks every four weeks as the MTD. However, after enrolling a total of 17 patients, we did not appreciate significant clinical activity (only two patients with stable disease) to warrant further expansion in this patient population. We have proved that the two drugs can be safely administered clinically at the aforementioned doses, and could be studied in other tumor types in the future. Temsirolimus appears to enhance the toxicities usually seen with irinotecan in terms of gastrointestinal and hematological adverse events. When the full dose of temsirolimus was given with low-dose weekly irinotecan, the regimen was not tolerated. The combination of irinotecan and temsirolimus did not meet the expectations of this study. However, in two patients this regimen stabilized the disease for about one year. This amounts to a 12% clinical benefit rate, which is similar to that published for mTOR inhibitors as single agent. One patient had a leiomyosarcoma, where alterations of the mTOR pathway have been described, such as the overexpression of RICTOR, a major component of the mTOR complex 2, which might contribute to the pathogenesis of well-differentiated leiomyosarcoma [38,39]. Unfortunately, there is no proven clinical biomarker to predict the activity of mTOR inhibitors against sarcomas. Thus further understanding of the role of the mTOR pathway in the oncogenesis of sarcomas is warranted. Prolonged stabilization of disease with a low toxicity regimen could be useful if the susceptible patients could be appropriately selected.

## References

[1] Laplante M., Sabatini D.M. (2012). mTOR signaling in growth control and disease. Cell.

[2] Wong K.K., Engelman J.A., Cantley L.C. (2010). Targeting the PI3K signaling pathway in cancer. Curr. Opin. Genet. Dev..

[3] Cully M., Downward J. (2009). Translational responses to growth factors and stress. Biochem. Soc. Trans..

[4] Sleijfer S., van der Graaf W.T., Blay J.Y. (2008). Angiogenesis inhibition in non-GIST soft tissue sarcomas. Oncologist.

[5] Falcon B.L., Barr S., Gokhale P.C., Chou J., Fogarty J., Depeille P., Miglarese M., Epstein D.M., McDonald D.M. (2011). Reduced VEGF production, angiogenesis, and vascular regrowth contribute to the antitumor properties of dual mTORC1/mTORC2 inhibitors. Cancer Res..

[6] Levine A.J., Feng Z., Mak T.W., You H., Jin S. (2006). Coordination and communication between the p53 and IGF-1-AKT-TOR signal transduction pathways. Genes Dev..

[7] Hudes G., Carducci M., Tomczak P., Dutcher J., Figlin R., Kapoor A., Staroslawska E., Sosman J., McDermott D., Bodrogi I. (2007). Temsirolimus, interferon alfa, or both for advanced renal-cell carcinoma. N. Engl. J. Med..

[8] Motzer R.J., Hudes G.R., Curti B.D., McDermott D.F., Escudier B.J., Negrier S., Duclos B., Moore L., O’Toole T., Boni J.P. (2007). Phase I/II trial of temsirolimus combined with interferon alfa for advanced renal cell carcinoma. J. Clin. Oncol..

[9] Motzer R.J., Escudier B., Oudard S., Hutson T.E., Porta C., Bracarda S., Grunwald V., Thompson J.A., Figlin R.A., Hollaender N. (2008). Efficacy of everolimus in advanced renal cell carcinoma: A double-blind, randomised, placebo-controlled phase III trial. Lancet.

[10] Pavel M.E., Hainsworth J.D., Baudin E., Peeters M., Horsch D., Winkler R.E., Klimovsky J., Lebwohl D., Jehl V., Wolin E.M. (2011). Everolimus plus octreotide long-acting repeatable for the treatment of advanced neuroendocrine tumours associated with carcinoid syndrome (RADIANT-2): A randomised, placebo-controlled, phase 3 study. Lancet.

[11] Baselga J., Campone M., Piccart M., Burris H.A., Rugo H.S., Sahmoud T., Noguchi S., Gnant M., Pritchard K.I., Lebrun F. (2012). Everolimus in postmenopausal hormone-receptor-positive advanced breast cancer. N. Engl. J. Med..

[12] Dickson M.A., Schwartz G.K., Antonescu C.R., Kwiatkowski D.J., Malinowska I.A. (2013). Extrarenal perivascular epithelioid cell tumors (PEComas) respond to mTOR inhibition: Clinical and molecular correlates. Int. J. Cancer.

[13] Dabora S.L., Franz D.N., Ashwal S., Sagalowsky A., DiMario F.J., Miles D., Cutler D., Krueger D., Uppot R.N., Rabenou R. (2011). Multicenter phase 2 trial of sirolimus for tuberous sclerosis: Kidney angiomyolipomas and other tumors regress and VEGF-D levels decrease. PLoS One.

[14] Okuno S., Bailey H., Mahoney M.R., Adkins D., Maples W., Fitch T., Ettinger D., Erlichman C., Sarkaria J.N. (2011). A phase 2 study of temsirolimus (CCI-779) in patients with soft tissue sarcomas: A study of the Mayo phase 2 consortium (P2C). Cancer.

[15] Chawla S.P., Staddon A.P., Baker L.H., Schuetze S.M., Tolcher A.W., D’Amato G.Z., Blay J.Y., Mita M.M., Sankhala K.K., Berk L. (2012). Phase II study of the mammalian target of rapamycin inhibitor ridaforolimus in patients with advanced bone and soft tissue sarcomas. J. Clin. Oncol..

[16] Chawla S., Blay J., Ray-Coquard L., Cesne A., Staddon A., Milhem M., Penel N., Riedel R., Nguyen B., Cranmer L. (2011). Results of the phase III, placebo-controlled trial (SUCCEED) evaluating the mTOR inhibitor ridaforolimus (R) as maintenance therapy in advanced sarcoma patients (pts) following clinical benefit from prior standard cytotoxic chemotherapy. J. Clin. Oncol..

[17] Creel P.A. (2009). Management of mTOR inhibitor side effects. Clin. J. Oncol. Nurs..

[18] Alagoz M., Gilbert D.C., El-Khamisy S., Chalmers A.J. (2012). DNA repair and resistance to topoisomerase I inhibitors: Mechanisms, biomarkers and therapeutic targets. Curr. Med. Chem..

[19] Jackson S.P. (2009). The DNA-damage response: New molecular insights and new approaches to cancer therapy. Biochem. Soc. Trans..

[20] Casey D.A., Wexler L.H., Merchant M.S., Chou A.J., Merola P.R., Price A.P., Meyers P.A. (2009). Irinotecan and temozolomide for Ewing sarcoma: The Memorial Sloan-Kettering experience. Pediatr. Blood Cancer.

[21] Pappo A.S., Lyden E., Breitfeld P., Donaldson S.S., Wiener E., Parham D., Crews K.R., Houghton P., Meyer W.H. (2007). Two consecutive phase II window trials of irinotecan alone or in combination with vincristine for the treatment of metastatic rhabdomyosarcoma: The Children’s Oncology Group. J. Clin. Oncol..

[22] Vassal G., Couanet D., Stockdale E., Geoffray A., Geoerger B., Orbach D., Pichon F., Gentet J.C., Picton S., Bergeron C. (2007). Phase II trial of irinotecan in children with relapsed or refractory rhabdomyosarcoma: A joint study of the French Society of Pediatric Oncology and the United Kingdom Children’s Cancer Study Group. J. Clin. Oncol..

[23] Bisogno G., Riccardi R., Ruggiero A., Arcamone G., Prete A., Surico G., Provenzi M., Bertolini P., Paolucci P., Carli M. (2006). Phase II study of a protracted irinotecan schedule in children with refractory or recurrent soft tissue sarcoma. Cancer.

[24] Eckhardt S.G. (1998). Irinotecan: A review of the initial phase I trials. Oncology.

[25] Pencreach E., Guerin E., Nicolet C., Lelong-Rebel I., Voegeli A.C., Oudet P., Larsen A.K., Gaub M.P., Guenot D. (2009). Marked activity of irinotecan and rapamycin combination toward colon cancer cells *in vivo* and *in vitro* is mediated through cooperative modulation of the mammalian target of rapamycin/hypoxia-inducible factor-1alpha axis. Clin. Cancer Res..

[26] Lee S.J., Gounder M., Rubin E.H., Li J.M., Gu Z., Thalasila A., Loyer E., Kudelka A.P., Verschraegen C.F. (2008). Optimal modeling for phase I design of a two drug combination-results of a phase I study of cisplatin with 9-nitrocamptothecin. Invest. New Drugs.

[27] Back J.H., Rezvani H.R., Zhu Y., Guyonnet-Duperat V., Athar M., Ratner D., Kim A.L. (2011). Cancer cell survival following DNA damage-mediated premature senescence is regulated by mammalian target of rapamycin (mTOR)-dependent Inhibition of sirtuin 1. J. Biol. Chem..

[28] Sabatini D.M., Erdjument-Bromage H., Lui M., Tempst P., Snyder S.H. (1994). RAFT1: A mammalian protein that binds to FKBP12 in a rapamycin-dependent fashion and is homologous to yeast TORs. Cell.

[29] Boni J.P., Hug B., Leister C., Sonnichsen D. (2009). Intravenous temsirolimus in cancer patients: Clinical pharmacology and dosing considerations. Semin. Oncol..

[30] Klumpen H.J., Beijnen J.H., Gurney H., Schellens J.H. (2010). Inhibitors of mTOR. Oncologist.

[31] Dilling M.B., Dias P., Shapiro D.N., Germain G.S., Johnson R.K., Houghton P.J. (1994). Rapamycin selectively inhibits the growth of childhood rhabdomyosarcoma cells through inhibition of signaling via the type I insulin-like growth factor receptor. Cancer Res..

[32] Ogawa T., Tokuda M., Tomizawa K., Matsui H., Itano T., Konishi R., Nagahata S., Hatase O. (1998). Osteoblastic differentiation is enhanced by rapamycin in rat osteoblast-like osteosarcoma (ROS 17/2.8) cells. Biochem. Biophys. Res. Commun..

[33] Mateo-Lozano S., Tirado O.M., Notario V. (2003). Rapamycin induces the fusion-type independent downregulation of the EWS/FLI-1 proteins and inhibits Ewing’s sarcoma cell proliferation. Oncogene.

[34] Schuetze S., Baker L., Maki R. (2006). Sirolimus reduced tumor-related morbidity and resulted in biochemical and radiographic response in patients with progressive sarcoma. J. Clin. Oncol..

[35] Mita M., Sankhala K., Abdel-Karim I., Mita A., Giles F. (2008). Deforolimus (AP23573) a novel mTOR inhibitor in clinical development. Expert Opin. Investig. Drugs.

[36] Garrido-Laguna I., Janku F. (2012). Ridaforolimus in advanced sarcomas: A leap forward or missed opportunity?. J. Clin. Oncol..

[37] Schuetze S.M., Zhao L., Chugh R., Thomas D.G., Lucas D.R., Metko G., Zalupski M.M., Baker L.H. (2012). Results of a phase II study of sirolimus and cyclophosphamide in patients with advanced sarcoma. Eur. J. Cancer.

[38] Gibault L., Ferreira C., Perot G., Audebourg A., Chibon F., Bonnin S., Lagarde P., Vacher-Lavenu M.C., Terrier P., Coindre J.M. (2012). From PTEN loss of expression to RICTOR role in smooth muscle differentiation: Complex involvement of the mTOR pathway in leiomyosarcomas and pleomorphic sarcomas. Mod. Pathol..

[39] Italiano A., Kind M., Stoeckle E., Jones N., Coindre J.M., Bui B. (2011). Temsirolimus in advanced leiomyosarcomas: Patterns of response and correlation with the activation of the mammalian target of rapamycin pathway. Anticancer Drugs.

